# Techno-Functional Performance of Emmer, Spelt and Khorasan in Spontaneously Fermented Sourdough Bread

**DOI:** 10.3390/foods11233927

**Published:** 2022-12-05

**Authors:** Dubravka Škrobot, Tamara Dapčević-Hadnađev, Jelena Tomić, Nikola Maravić, Nikola Popović, Pavle Jovanov, Miroslav Hadnađev

**Affiliations:** 1 Institute of Food Technology, University of Novi Sad, Bulevar Cara Lazara 1, 21000 Novi Sad, Serbia; 2 Institute of Molecular Genetics and Genetic Engineering, University of Belgrade, Vojvode Stepe 9, 444a, 11042 Belgrade, Serbia

**Keywords:** ancient wheat, bread quality, dough rheology, sourdough, spontaneous fermentation

## Abstract

The aim of this study was to test the suitability of three different ancient wheat varieties (emmer, spelt and khorasan) to produce spontaneously fermented sourdough bread and to evaluate the impact on the dough rheological properties, ultrastructure and baking quality. Modern wheat sourdough bread and bakery yeast fermented bread were used as controls. Sourdoughs produced from modern and ancient wheats exerted different effects on dough viscoelastic properties, bread specific volume, texture, firming rate, colour and sensory properties, while there was no influence on bread water activity. Both khorasan sourdough, being characterised with the highest dough strength and dense gluten protein matrix, and emmer sourdough, with loose and thin gluten strands of low strength, yielded breads characterised by low specific volume and hard crumb texture. Spelt and modern wheat sourdough were characterised by foam-like dough structures with entrapped gas cells leading to breads of similar specific volume and texture. Although the yeast-fermented wheat flour exerted a higher specific volume and the lowest firmness, the sourdough wheat flour bread had a lower firming rate. A comparison of sourdough bread prepared with modern and ancient wheats revealed that breads based on ancient varieties possess a less noticeable sour taste, odour and flavour, thus contributing to more sensory-appealing sourdough bread.

## 1. Introduction

The paradigm of agricultural development focusing narrowly on increasing crop yields has caused clear-cut homogeneity in the global cereal supply and obvious dominance of three major cereal crops: wheat, rice and maize. While great strides have been made toward breeding new wheat varieties, genetic evolution has caused the creation of more productive plants with modified chemical compositions and other quality attributes. Paradoxically, improvements in grain yield, as well as some technological quality attributes of flour, has caused an increase in starch content and has consequently led to the reduction of other grain components, especially minerals [1]. The current over-dependence on a few types of crops could exacerbate many difficulties in food security, nutrition and health, making the food supply chain extremely vulnerable. Recognising the urgent need for diversification, there is an attempt to widen the range of crops for daily human consumption. Regarding future trends, demands for health-promoting foods will shift toward alternative and underutilised crops depending on the nutritional and techno-functional quality and climatic adaptability. In that sense, the ancient wheat varieties have received undoubted interest because of their combination of nutritional and functional attributes. Moreover, ancient wheat varieties with exceptional climate adaptability are convenient for low-input and organic farming, with a positive impact on biodiversity protection, thus possessing the great potential to become more competitive than some of the currently grown crops [2]. Regarding nutritional quality, ancient wheat varieties are comparable or superior to conventional ones (modern wheat), particularly regarding minor components present in grains (proteins, dietary fibre, resistant starch, minerals, vitamins and phenolic compounds) [1,3,4,5]. In recent years, there has been an effort by the research community to introduce ancient wheat flour into various bakery products, but the main drawbacks (poor rheology, problems with dough processing, lower quality of final product) imply that additional actions are still needed to overcome the above-mentioned shortcomings and further reveal the full potential of these underutilised wheats [6,7]. To achieve these goals, considerable technological improvements in the bakery industry are necessary and sourdough fermentation, as a sustainable food process, is recognised as a potential solution.

Sourdough fermentation, a mixture of water and flour fermented with lactic acid bacteria and yeasts, is a specific process where developed microbial communities are bound by a range of competitive and cooperative interactions that, through complex biochemical changes, result in the transformation of ingredients. These transformations, caused by complex microbiome consortiums, lead to positive nutritional implications in terms of increasing mineral, protein and free amino acid bioavailability, reducing anti-nutrients (phytic acid, tannins and saponins) and reducing starch digestibility, followed by a decrease in the glycaemic index of baked goods [8]. Among the advantages offered by sourdough fermentation is the improvement of sensory, rheology and shelf-life properties of baked goods [9]. Due to the benefits its offers in terms of nutrition, sustainability, plasticity for innovation, cultural heritage and strong consumer interest, sourdough technology is an excellent vector for food innovations. Since the biodiversity of microbiota is highly influenced by the nature of used raw materials, ancient wheat flours are quite original ecological niches that represent a true opportunity to increase both cereal and microbial biodiversity for food processing. 

There are a certain number of publications concerning the use of sourdough fermentation for the production of ancient wheat flour-based products [10,11,12]. However, according to our knowledge, the application of sourdough fermentation on ancient wheat flour is largely based on the addition of different starter cultures. Therefore, the objective of this study was to evaluate the effect of spontaneous sourdough fermentation on the rheological properties of dough and the quality of bread made from ancient wheat varieties (emmer, spelt and khorasan) in comparison with modern wheat. Moreover, the obtained bread samples were compared with bread prepared without sourdough but produced using a conventional baking procedure that includes the utilisation of bakery yeast to induce fermentation.

## 2. Materials and Methods

### 2.1. Materials

Three flours from three different ancient wheat varieties—emmer, spelt and khorasan—were purchased from a small local producer (Poljoprivredno Gazdinstvo Spelta Jevtić, Bačko Gradište, Serbia), whereas flour from a modern wheat variety was obtained from a milling company Danubius d.o.o. (Novi Sad, Serbia). All wheat samples were cultivated in the same production year and at the same location. After harvesting, grains were stored for six months. Subsequently, they were dehulled by Heger’s large-scale friction de-huller (Herrenberg, Germany) and milled by a large-scale stone mill Osttiroler Getreidemühlen (Dölsach, Austria). The obtained flour samples were as follows: modern wheat flour (moisture 11.2%, ash 1.48%, total fibre 10.45%, protein 11.3%), spelt flour (moisture 10.8%, ash 1.98%, total fibre 10.1%, protein 15.87%), emmer flour (moisture 11.31%, ash 1.87%, total fibre 9.58%, protein 15.99%) and khorasan flour (moisture 10.7%, ash 1.50%, total fibre 9.85%, protein 11.49%). The salt and bakery yeast used in the breadmaking were purchased from the local market.

### 2.2. Sourdough Preparation

Sourdough fermentation of modern wheat, emmer, spelt and khorasan flours was carried out without using starter cultures or baker’s yeast, through a back-slopping procedure (every 24 h, 5 days). Doughs were prepared by mixing flour and demineralised water (1:1 (*w*/*w*)) with a resulting dough yield of 200 and incubated at 25 °C for 24 h in a laboratory incubator (Friocell 111, MMM Medcenter Einrichtungen GmbH, München, Germany). After this first fermentation, back-slopping steps were further carried out by using the same condition of fermentation (25 °C for 24 h), where a portion of fermented dough from a previous day was used as an inoculum for subsequent back-slopping, mixed with flour and demineralised water in a 1:2:2 ratios (fermented dough:flour:water). Mature sourdough that was prepared as described above served as a “starter” instead of bakery yeast in the breadmaking procedure.

### 2.3. Sourdough Rheology

Sourdough rheological behaviour was monitored using a Haake Mars rheometer (Thermo Scientific, Karlsruhe, Germany). Frequency sweep measurements were carried out at 25 °C using PP35 S serrated parallel plate measuring geometry (35-mm diameter, 1-mm gap) to eliminate sample slippage during the tests. The excess of dough sample was removed and the edges were covered with paraffin oil to prevent the dough from drying during the measurement procedure. After that, samples were left to rest between the plates for 10 min, allowing residual stresses to relax. Mechanical spectra were recorded over the frequency range 1–10 Hz at a constant shear stress of 0.5 Pa, which was within the previously determined linear viscoelastic region. All tests were performed in triplicate.

### 2.4. Scanning Electron Microscopy (SEM)

Dough ultrastructure characterisation was carried out using a JEOL JSM 6460LV scanning electron microscope (Tokyo, Japan). All samples were previously lyophilised using an Alpha 1-2 LDplus freeze dryer (Martin Christ, Osterode am Harz Germany). Before scanning electron microscopy, powdered samples were mounted on scanning electron microscope stubs using double-sided adhesive tape and afterward coated with gold using Sputter Coater SC 005 (Bal-Tec GmbH, Schalksmühle, Germany). Consequently, the ultrastructure was analysed under high vacuum conditions at an accelerating voltage of 25 kV at a magnification of 1000×.

### 2.5. Breadmaking Procedure and Bread Evaluation

Control bread formulation consisted of 300 g of modern wheat flour, demineralised water up to 400 BU consistency, 7.5 g of bakery yeast and 5.4 g of salt. In doughs prepared with sourdough as a leavening agent, 25% of flour was replaced with the appropriate type of sourdough in order to keep the total flour amount at 300 g. Subsequently, 5.4 g of salt and demineralised water in an amount to achieve 400 BU consistency were added. Mixing was conducted in a Farinograph mixing bowl (Brabender Technologie GmbH & Co. KG, Duisburg, Germany) for seven minutes. Subsequently, dough samples were left for fermentation in a cabinet at 30 °C for 30 min and then punched down. This step was repeated for an additional 3 times and then the amount of 120 g of dough samples was hand-moulded and placed into Teflon pans (L × W × H: 80 mm × 50 mm × 50 mm). The proofing procedure was performed at 30 °C and a relative humidity of 85% up to the optimum volume increase (~3 h for samples containing sourdough and 65 min for control bread with bakery yeast). Consequently, the baking procedure was carried out in a modular deck oven (MD, Macpan SNS, Thiene, Italy) at 220 °C until a mass loss of 8%. Finally, bread samples were left to cool down for 2 h at room temperature and sealed in polyethylene bags for further analysis. Two batches of each bread sample were prepared.

### 2.6. Bread Volume and Water Activity Estimation

The specific volume of bread samples was measured on 3 loaves per batch by a Volscan Profiler (Stable Micro Systems, Godalming, UK). 

Water activity (a_w_) of bread crumb samples (5 mm thickness) taken from the middle of each bread loaf after 24 h of storage was measured by a LabSwift-a_w_ instrument (Novasina AG, Lachen, Switzerland).

### 2.7. Texture Measurements

Breadcrumb textural properties were determined at room temperature by TA.XT2 Texture Analyser (Stable Micro Systems, Godalming, UK) equipped with a 30-kg load cell. Measurements were performed using the modified standard method for the determination of bread firmness AACC (74-09) [13]. The modification involved using a P/0.5 inch diameter cylinder probe instead of a P/36 mm probe due to the smaller bread volume in comparison with a standard bread loaf. Breadcrumb firmness was determined on 3 slices (10 mm thickness) from the middle of each bread loaf (3 loaves per batch) in a compression mode at 1 mm/s pre-test speed, 1.7 mm/s test speed and 10 mm/s post-test speed and at 40% strain 24 h, 48 h and 72 h after baking.

### 2.8. Colour Measurements

Bread crust and bread crumb colour was evaluated in six replicates per loaf, 24 after baking using a Minolta Chroma Meter CR-400 colorimeter (KonicaMinolta Sensing Inc., Osaka, Japan) (8 mm Ø contact area). Prior to the measurements, the instrument was calibrated by a standard light white reference tile and the tests were performed under standard illuminant D65. The obtained results were expressed according to the CIELab colour system (L*—lightness; a*—redness to greenness, positive to negative values, respectively; b*—yellowness to blueness, positive to negative values, respectively).

### 2.9. Sensory Analysis

Sensory profiling was performed on bread samples through quantitative descriptive analysis QDA [14] by a FINS (Institute of Food Technology in Novi Sad) trained panel (*n* = 10, 70% female, 30% male, average age 29 years). The descriptive terminology of the products was created in a pre-trial session using a control and bread from emmer flour. After two 1 h pre-trial sessions, the descriptors, together with their definitions and reference samples, were agreed upon by the panellists. The final list encompassed five appearance attributes (crust colour, crumb colour, pore size min, pore size max and pore uniformity), three taste attributes (saltiness, sourness and bitterness), eight odour attributes (overall intensity, cheese, yeast, flour, vinegar, wet grains, malt, toasted), eight flavour attributes (overall intensity, cheese, yeast, flour, vinegar, flavour persistence, malt, toasted) and five textural attributes (hardness, sharpness, moisture absorption, adhesiveness, crumb compactness). Once the descriptors had been selected, panellists were trained by measuring the intensities of selected descriptors perceived in breads using 10-cm unstructured scales. By the end of the pre-trial sessions, all panellists were able to discriminate among samples with good repeatability between sessions and to reach agreement with other assessors in the panel. 

The sensory analysis was conducted in individual sensory booths. The bread samples (one slice per sample) were served at room temperature in closed odourless plastic containers coded with 3-digit random numbers following a complete balance block design. All panellists evaluated all samples in a sequential monadic manner. The sensory profiling was done in two repetitions. Water was used for rinsing between samples.

### 2.10. Consumer Test

A consumer test was performed with 72 consumers (45 female and 27 male, aged between 20 and 45 years) recruited from students and academic staff from the University of Novi Sad based on bread consumption frequency (at least 3 times per week) and without food allergies, especially celiac and gluten sensitivity. Consumers were asked to rate how much they liked the bread samples in terms of overall acceptability, the taste, the texture of the crumb and the texture of the crust using the 9-point hedonic scale with the extremes labelled 1 = dislike very much and 9 = like very much. The bread samples were distributed in closed odourless plastic containers coded with 3-digit random numbers. The order of sample presentation was randomised across consumers. The evaluation was performed in individual sensory booths. 

This study was approved by the Ethics Committee of the Institute of Food Technology in Novi Sad, University of Novi Sad, Serbia (No. 175/I/7-3).

### 2.11. Statistical Analysis

All measurements were performed in replicate and the mean values ± standard deviations were reported. One-way analysis of variance (ANOVA) followed by Tukey’s minimum square difference test was carried out for the evaluation of statistical differences between tested samples using Statistica 10.0 (StatSoft Inc., Tulsa, OK, USA), whereas XLSTAT 2022.1.2 (Addinsoft) was used for sensory and consumer data analysis. The significance of the difference between groups was indicated at the 95% confidence level.

## 3. Results and Discussion

### 3.1. Rheological Behaviour of Sourdough Samples

**Figure 1:**
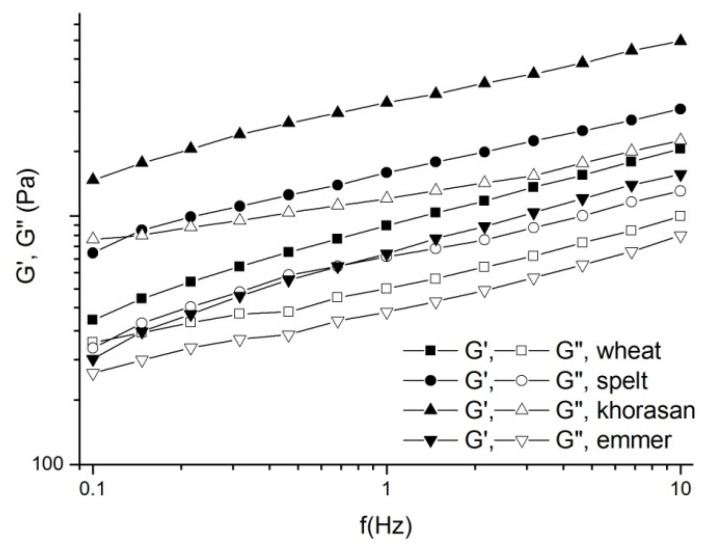
Mechanical spectra of mature wheat, emmer, khorasan and spelt sourdough.

The effect of wheat variety on the viscoelastic properties of spontaneously fermented sourdough was assessed using dynamic oscillatory measurements. According to obtained mechanical spectra (Figure 1), storage modulus G’ was higher than loss modulus G” in the examined frequency range, indicating the solid elastic-like behaviour of sourdoughs. The prevalence of elastic properties over viscous has already been reported for sourdough samples [15,16]. Values of tan δ (G”/G’) were in the following range: 0.42, 0.47, 0.56 and 0.58 for khorasan, spelt, wheat and emmer sourdough, respectively. Among sourdough samples, khorasan sourdough had the highest values of modulus G’ and the lowest values of tan δ, thus exhibiting the properties of rigid and stiff material. This behaviour could be related to the lower wet gluten content and higher gluten index value [17] of khorasan sourdough in comparison with other samples, resulting in a higher amount of elastic over viscous gluten components. On the contrary, emmer sourdough exhibited the lowest elastic modulus and the highest tan δ values, suggesting that emmer flour produced sourdough with the lowest strength and elasticity compared with other wheat varieties. This was in agreement with the fact that emmer flour was characterised with the lowest gluten index values in comparison with spelt and khorasan flour [17] and therefore resulted in a higher contribution of viscous over elastic gluten component in the measured sourdough system.

### 3.2. Scanning Electron Microscopy of Sourdough

**Figure 2:**
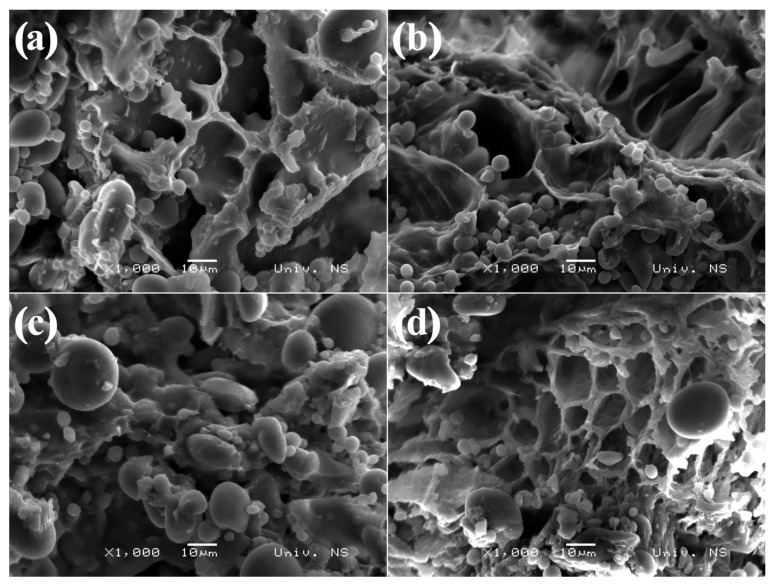
Scanning electron micrographs of (**a**) modern wheat, (**b**) emmer, (**c**) khorasan and (**d**) spelt sourdough samples at 1000× magnification.

Sourdough ultrastructure is illustrated in Figure 2.

All sourdough samples contained small and large starch granules of spherical shape distributed along the protein matrix, where khorasan and spelt sourdough samples were distinguished by the higher number of the large starch granules. Khorasan sourdough was characterised by a more compact protein network compared with the other sourdoughs, with starch granules embedded in the dense gluten protein matrix without noticeable cavities corresponding to CO_2_ bubbles. These results are in accordance with results of rheological characterisation, where khorasan sourdough exhibited the highest dough strength and elasticity (Figure 1). On the contrary, other sourdough samples exhibited foam-like structures with entrapped gas cells which could be related to gas production activity and alcoholic and other compounds generated during fermentation. While in the modern wheat and spelt sourdough the gluten network strands surrounding the cell walls were thick, the emmer sourdough was characterised by loose and thin strands that collapsed upon gas cell expansion during fermentation, resulting in slightly elongated pores.

### 3.3. Bread Volume, Water Activity and Texture

The studies investigating the effect of sourdough addition on bread technological performance are quite contradictory. Depending on flour type, sourdough type, LAB strain, fermentation conditions, etc., sourdough fermentation can influence both bread volume increase and texture softening effect [18] and vice versa [19,20]. Microbial activity and the types of microbial metabolites produced affect protein structural and conformational changes, leading to gluten complex hydrolysis, swelling and increased solubility [21]. Simultaneously, the activity of alpha-amylase is altered, influencing changes in the moisture absorption of polysaccharides and eventually degradation of the cross-linked gluten proteins that are responsible for bread firmness [22]. This is all supported by exopolysaccharides produced by some LAB strains that exhibit structure forming ability thus affecting both bread volume and structure [21].

The results obtained in this study revealed that all investigated sourdough containing samples exhibited lower specific volume in comparison with the control sample, due to the expressed activity of bakery yeast and superior gluten properties of modern wheat variety (Table 1). According to Abedfar and Sadeghi [22], specific volume is generally influenced by the quantity of CO_2_ produced in the fermentation process as well as the gas retention properties of the dough. Among sourdough breads, wheat and spelt samples were characterised with the highest specific volume, while bread prepared from khorasan sourdough and flour had the lowest specific volume. This could be related to the lowest protein content (khorasan, 11.49%; spelt, 15.87%; emmer, 15.99%) and the wet gluten value of khorasan flour in comparison with other ancient wheat varieties [17], as well as the low quantity of CO_2_ produced in the fermentation step. Moreover, khorasan sourdough was characterised by high stiffness (Figure 1) and dense structure (Figure 2), which could not expand enough to entrap formed gas bubbles. Although spelt and emmer flour were characterised by a higher protein content, breads prepared with those flours had lower specific volumes in comparison with the bread samples based on modern wheat flour and sourdough. Previous investigations by Bojnanska and Francakova [23], Boukid et al., [24] and Sumczynski et al., [25] also revealed that ancient wheat varieties were generally characterised by higher protein content than the modern wheats. However, it was also found that the gluten structure of these proteins was weak due to higher gliadin/glutenin ratios in comparison with the modern wheat variety [17,26].

Water represents one of the most important parameters influencing microbial spoilage of foods; the water activity (a_w_) factor is a valuable tool for predicting microbial growth and metabolic activity [27]. The obtained results revealed that all breadcrumb samples were characterised by relatively high a_w_ values that did not significantly differ among themselves. The obtained a_w_ values are in accordance with the findings of Neylon et al., [28], who obtained a_w_ values of tested bread crumb samples in the range of 0.95–0.97.

Crumb texture represents one of the most important parameters in bread quality monitoring. According to the obtained results (Table 2), it can be concluded that all sourdough-based breads expressed higher crumb firmness value in comparison with the control sample. Moreover, the firmness value of the sample prepared with modern wheat sourdough and flour was the lowest, while it was the highest for the khorasan sourdough bread. These results are in agreement with specific volume values. Namely, the samples that were characterised by a higher specific volume exhibited lower firmness values and vice versa. According to Goesaert et al., [29] and Skendi et al., [30], breadcrumb hardness is generally influenced by bread volume as well as the density of bread loaves as a consequence of total gas cell area increase. Moreover, during the storage period, all samples showed an increase in breadcrumb firmness values, which is an indicator of bread staling [31]. The increase in breadcrumb firmness could be related to moisture loss and the starch retrogradation effect [32]. During a storage period from 24 h to 72 h, the increase in breadcrumb firmness was 10.2%, 8.3%, 15.2%, 19.2% and 39.2% for the control, wheat, emmer, khorasan and spelt bread, respectively. Comparing yeast and sourdough wheat fermented bread, the sourdough fermentation led to a lower crumb firming rate, which is in agreement with the findings of Novotni et al., [33], who concluded that a *Lactobacillus plantarum* sourdough addition most efficiently retarded the firming rate of wholemeal wheat flour bread in comparison with partially baked frozen bread without sourdough. However, sourdough breads prepared with ancient wheat varieties exhibited a faster firming rate than modern wheat sourdough bread. Among ancient wheat varieties, emmer sourdough bread, being characterised with the weakest dough structure and prevalence of viscous over elastic behaviour (Figure 1), exhibited the lowest firming rate.

### 3.4. Bread Colour and Appearance

**Figure 3:**
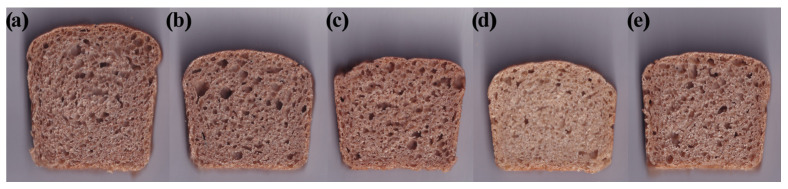
Bread crumb appearance of (**a**) control (yeast fermented), (**b**) wheat, (**c**) emmer, (**d**) khorasan and (**e**) spelt sourdough bread.

Cross-sectional views of breadcrumb samples are summarised in Figure 3. It can be observed that the khorasan sourdough was characterised by the densest and most uniform structures, followed by the control bread. All sourdough-containing samples, except khorasan, had non-uniform pore structures with large pore cell sizes, which is a characteristic of sourdough bread [18].

The crusts and crumbs of bread samples prepared with wheat, emmer and spelt sourdough and flour showed lower L* values, namely they were darker, whilst khorasan bread crusts and crumbs were significantly lighter (Table 3). Moreover, the b* value indicating the yellowness of the samples was also the most pronounced for both the crusts and the crumbs of the khorasan breads. This could be related to the high carotenoid pigment found in khorasan flour. Pasqualone et al. [34] determined that khorasan flour had the highest carotenoid content in comparison with durum, spelt and wheat flour, resulting in more yellowish breads. However, a decrease in bread crust lightness and an increase in a* values of the rest of the sourdough-containing samples in comparison with the control could be influenced by a more intense Maillard browning reaction in breads containing sourdough. Namely, Gänzle [35] revealed that the sourdough fermentation of wheat and rye results in an increased activity of amylase, which influences starch hydrolysis and maltodextrins and maltose and glucose development, thus influencing more intense browning reactions in the bread-baking step.

### 3.5. Sensory Analysis of Bread

**Figure 4:**
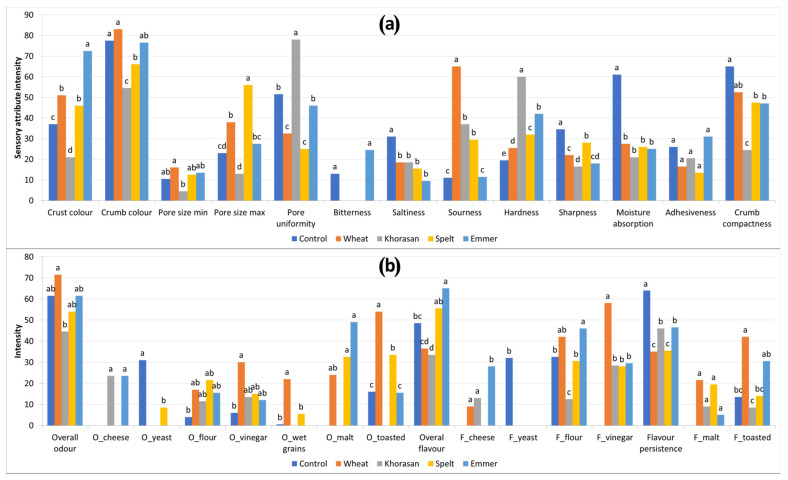
Average intensities of the appearance and texture (**a**) and odour and flavour (**b**) of sourdough bread samples (“O” in the name of sensory descriptor refers to the “odour” attribute, while “F” refers to the “flavour” attribute) (different letters above the bars indicate statistical differences (*p* < 0.05) among the samples according to Tukey’s minimum square difference test).

According to ANOVA, almost all selected sensory attributes help to discriminate sensory profiles among the samples (Figure 4). The main differences among the samples were related to the textural, odour and flavour properties. Sourdough breads showed a more complex odour and flavour profile in comparison with the control bread. This may be the result of the specific interaction between the flour and the microbial strains that participated in the fermentation process, followed by complex biochemical transitions that occurred during baking. It is well documented that sourdough fermentation accelerates the formation of more diverse volatile compounds in bread compared with bread fermented only with bakery yeast as a consequence of the different metabolic pathways of sourdough microbiota and bakery yeast [36,37,38]. During baking, the Maillard reaction produces other volatile compounds that contribute to the typical flavour in the crust [39]. Regarding the crust colour, all sourdough breads, except the khorasan bread, were darker than the control, which is consistent with the results of the instrumentally measured colour. The bread with the khorasan flour was the most distinct sample, with a significantly (*p* < 0.05) lighter colour of crumb and crust and smaller sized uniform pores with a more compact and hard crumb texture than all the other samples. Moreover, although overall odour and flavour of this bread were mild, it was specific and intense enough to allow a clear distinction of this sample in terms of cheese, flour and vinegar odour and flavour notes. The bread with emmer was similar to the khorasan bread in terms of cheesy odour and flavour, while, in terms of textural attributes, significant differences among them were observed for hardness and crumb compactness. Moreover, the bread with emmer possessed a significantly (*p* < 0.05) darker crust colour, odour on malt, bitter taste and overall flavour intensity in comparison with all the other samples. The control bread stood out from the other samples with the more noticeable sharpness and moisture absorption, odour and flavour on yeast, as well as flavour persistence, which is consistent with the recipes and the experimental design. All the sourdough bread samples were more sour than the control bread, with the exception of emmer bread, which was perceived as equally sour as the control. The sourdough bread made with modern wheat flour was described as significantly (*p* < 0.05) more sour, with a very intense odour and flavour on vinegar. A similar finding was reported by Coda et al., [10], who found that bread with spelt and white wheat flour showed a higher acidic flavour compared with the emmer sourdough bread.

### 3.6. Consumer Test

**Figure 5:**
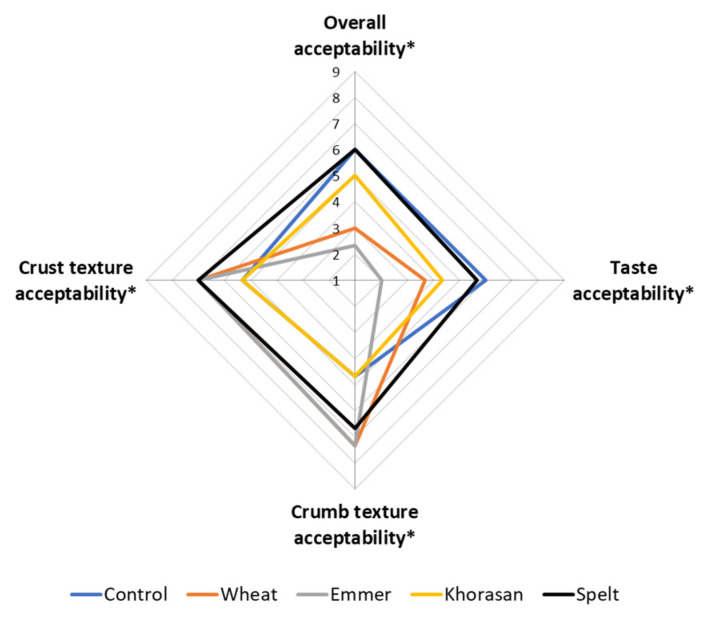
Spider plot of the sensory acceptance (overall, taste, crumb and crust texture) given by the consumers for the bread samples (* indicate significant (*p* < 0.05) difference in acceptability between the bread samples according to the Tukey’s minimum square difference test).

To create a visual representation of the sensory acceptability (overall bread acceptability, acceptability of the bread taste and crumb and crust texture) of the bread samples given by the participants involved in the consumer study, a spider plot was created (Figure 5) by plotting mean intensity values on a corresponding scale (1 = dislike very much and 9 = like very much). Individual sensory attributes that were evaluated are positioned as the spokes around a central point where the spokes represent the attribute intensity scale, with more intense values radiating outside. Bread samples were considered acceptable if their mean scores were above 5 (neither like nor dislike) and the plot illustrates that mean values for all examined sensory properties for the control and the spelt bread were above the defined threshold of 5.0. This indicates that consumers on average liked the control and the spelt bread more than the others. The lower liking of the emmer and the wheat sourdough bread is probably due to a slightly lower liking of taste, which is recognised as one of the key sensory attributes affecting the product’s overall acceptability [40]. On the other hand, the khorasan bread was the least liked in terms of crumb and crust texture. This could be due to the absence of large pores and the characteristic airy porous structure of the breadcrumbs observed by the trained sensory panel (Figure 4a). The appearance and the tactile perception of the bread texture are highly relevant criteria for consumers’ acceptability [41]. The crumbs’ feel by touch or in the mouth is greatly influenced by the size or the structure of the crumb cells. The compact and dense structure of the khorasan bread contributes to a firmer and more rigid texture with coarse and thick-walled cell structures, which finally results in the lowest score of textural acceptability.

## 4. Conclusions

The suitability of three different ancient wheat varieties (emmer, spelt and khorasan) to produce spontaneously fermented sourdough bread was evaluated by comparing their dough rheological properties, ultrastructure and breadmaking quality with modern wheat bread produced from sourdough, as well as with bread produced using bakery yeast without sourdough addition. A khorasan sourdough and an emmer sourdough yielded breads with low specific volume and hard crumb texture. On the contrary, spelt sourdough, characterised by a foam-like dough structure and balanced dough viscoelasticity, produced bread of similar specific volume and texture as modern wheat sourdough. Although the bread prepared from bakery yeast had a superior quality in terms of specific volume and texture, the sourdough bread from the modern wheat flour was characterised by a lower firming rate. The comparison between modern and ancient wheat varieties revealed that sourdough bread from ancient wheats was perceived less sour in taste, odour and flavour, thus producing a more sensory appealing product than modern wheat. In addition, consumer tests indicated that overall acceptability was the highest for the yeast-fermented and spelt sourdough bread, followed by khorasan, wheat and emmer sourdough bread, respectively. In general, the obtained results indicated that it is possible to produce technologically and sensory acceptable sourdough bread from ancient wheat varieties and thus contribute to both cereal and microbial biodiversity protection. Future work can be oriented towards investigations of the potential of mixtures of different ancient wheat varieties for sourdough bread production and quality improvement.

## Figures and Tables

**Table 1:** Specific loaf volumes and crumb moisture activity (a_w_) values of sourdough breads prepared from different wheat varieties in comparison with control yeast fermented wheat bread.

Bread Sample	Specific Volume (mL/g)	a_w_ Value
Control	2.30 ± 0.072 ^d^	0.949 ± 0.0007 ^a^
Wheat	1.83 ± 0.020 ^c^	0.946 ± 0.0021 ^a^
Emmer	1.69 ± 0.033 ^b^	0.942 ± 0.0035 ^a^
Khorasan	1.52 ± 0.019 ^a^	0.944 ± 0.0002 ^a^
Spelt	1.81 ± 0.025 ^c^	0.948 ± 0.0001 ^a^

Values representing the mean ± standard deviation followed by the different letter in the column are not significantly different according to Tukey’s minimum square difference test (*p* < 0.05).

**Table 2:** Breadcrumb firmness and its evolution during storage of sourdough breads prepared from different wheat varieties in comparison with control yeast fermented wheat bread.

Bread Sample	Firmness (g)		
	24 h	48 h	72 h
Control	283.2 ± 4.04 ^aA^	295.6 ± 33.93 ^abA^	312.2 ± 28.08 ^bA^
Wheat	473.3 ± 4.07 ^aB^	491.3 ± 33.67 ^abB^	512.5 ± 53.24 ^bB^
Emmer	679.7 ± 61.47 ^aD^	702.9 ± 70.18 ^aC^	783.3 ± 60.70 ^bC^
Khorasan	910.4 ± 81.76 ^aE^	972.5 ± 57.39 ^bD^	1084.9 ± 45.57 ^cD^
Spelt	577.7 ± 51.50 ^aC^	707.9 ± 60.61 ^bC^	804.4 ± 71.76 ^cC^

Values representing the mean ± standard deviation. Different small letters above the values within the row and different capital letters within the column above the values indicate significant difference according to Tukey’s minimum square difference test (*p* < 0.05).

**Table 3:** Crust and crumb colour of yeast fermented (control) and wheat, emmer, khorasan and spelt sourdough bread.

Bread Sample	Crust			Crumb		
	L*	a*	b*	L*	a*	b*
Control	49.50 ± 1.506 ^c^	11.05 ± 0.313 ^a^	23.38 ± 0.275 ^bc^	48.33 ± 1.595 ^ab^	6.58 ± 0.301 ^ab^	16.10 ± 0.516 ^a^
Wheat	43.81 ± 1.010 ^a^	13.63 ± 0.465 ^b^	22.10 ± 0.582 ^ab^	47.05 ± 0.969 ^a^	6.67 ± 0.157 ^ab^	16.09 ± 0.357 ^a^
Emmer	42.69 ± 0.575 ^a^	14.82 ± 0.177 ^b^	21.83 ± 1.049 ^a^	48.54 ± 0.311 ^ab^	7.15 ± 0.230 ^b^	17.16 ± 0.526 ^ab^
Khorasan	55.08 ± 1.093 ^d^	11.49 ± 1.676 ^a^	27.98 ± 1.355 ^d^	58.46 ± 0.994 ^c^	5.28 ± 0.256 ^a^	19.48 ± 0.528 ^c^
Spelt	47.09 ± 0.755 ^b^	13.57 ± 0.356 ^b^	23.83 ± 0.533 ^c^	49.66 ± 2.527 ^b^	7.30 ± 2.826 ^b^	17.85 ± 1.637 ^b^

Values representing the mean ± standard deviation followed by the different letter in the column are significantly different according to Tukey’s minimum square difference test (*p* < 0.05).

## Data Availability

Data is contained within the article.
